# Lipidomic analysis reveals metabolism alteration associated with subclinical carotid atherosclerosis in type 2 diabetes

**DOI:** 10.1186/s12933-025-02701-z

**Published:** 2025-04-02

**Authors:** Maria Barranco-Altirriba, Joana Rossell, Núria Alonso, Ralf J. M. Weber, Emilio Ortega, Gavin R. Lloyd, Marta Hernandez, Oscar Yanes, Jordi Capellades, Catherine Winder, Alexandra Junza, Mireia Falguera, Josep Franch-Nadal, Warwick B. Dunn, Alexandre Perera-Lluna, Esmeralda Castelblanco, Didac Mauricio

**Affiliations:** 1 Department of Endocrinology & Nutrition, Hospital de la Santa Creu i Sant Pau, Barcelona, Spain; 2 B2SLab, Department of Systems Engineering, Automatics, and Industrial Informatics, Universitat Politècnica de Catalunya, Barcelona, Spain; 3 Networking Biomedical Research Centre in the Subject Area of Bioengineering, Biomaterials and Nanomedicine (CIBER-BBN), Instituto de Salud Carlos III (ISCIII), Madrid, Spain; 4 Institut de Recerca Sant Joan de Déu, Esplugues de Llobregat, Barcelona, Spain; 5 CIBER of Diabetes and Associated Metabolic Diseases (CIBERDEM), Instituto de Salud Carlos III (ISCIII), 28029 Madrid, Spain; 6 Institut d’Investigació Biomèdica Sant Pau (IR-Sant Pau), 08041 Barcelona, Spain; 7 Department of Endocrinology and Nutrition, Hospital Universitari i Institut d’Investigació en Ciències de la Salut Germans Trias i Pujol, 08916 Badalona, Spain; 8 Faculty of Medicine, Universitat Autònoma de Barcelona (UAB), Barcelona, Spain; 9 School of Biosciences, University of Birmingham, Edgbaston, Birmingham, B15 2TT UK; 10 Phenome Centre Birmingham, University of Birmingham, Edgbaston, Birmingham, B15 2TT UK; 11 Institute of Metabolism and Systems Research, University of Birmingham, Edgbaston, Birmingham, B15 2TT UK; 12 Department of Endocrinology and Nutrition, Institut d’Investigacions Biomèdiques August Pi Sunyer (IDIBAPS), Hospital Clinic, 08036 Barcelona, Spain; 13 Biomedical Research Networking Center for Physiopathology of Obesity and Nutrition (CIBEROBN), Instituto de Salud Carlos III (ISCIII), Madrid, Spain; 14 Department of Endocrinology and Nutrition, Hospital Universitari Arnau de Vilanova i Institut d’investigació biomèdica de Lleida (IRBLleida), Lleida, Spain; 15 Department of Electronic Engineering, Universitat Rovira i Virgili, IISPV, Tarragona, Spain; 16 Institute of Health Research Pere Virgili (IISPV), Tarragona, Spain; 17 Department of Biochemistry, Cell and Systems Biology, Centre for Metabolomics Research, Institute of Systems, Molecular and Integrative Biology, University of Liverpool, Liverpool, L69 7ZB UK; 18 Scientific and Technical Resources Services, Universitat Rovira i Virgili, Tarragona, Spain; 19 Institute for Biomedical Research Dr. Pifarré Foundation IRB Lleida, University of Lleida and Primary Health Care Centre Tàrrega, Gerència d’Atenció Primaria, Institut Català de la Salut, Lleida, Spain; 20 DAP-Cat Group, Research Support Unit, Institut Universitari d’Investigació en Atenció Primària Jordi Gol, Barcelona, Spain; 21 Division of Endocrinology, Metabolism and Lipid Research, Department of Internal Medicine, Washington University School of Medicine, St. Louis, MO 63110 USA; 22 Research Support Unit, Institut Universitari d’Investigació en Atenció Primària Jordi Gol i Gurina, 08007 Barcelona, Spain; 23 Faculty of Medicine, Universitat de Vic - Central University of Catalonia, Vic, Spain

**Keywords:** Lipidomic profile, Type 1 diabetes, Type 2 diabetes, Subclinical carotid atherosclerosis, Smoking habit

## Abstract

**Background:**

Disruption of lipid metabolism contributes to increased cardiovascular risk in diabetes.

**Methods:**

We evaluated the associations between serum lipidomic profile and subclinical carotid atherosclerosis (SCA) in type 1 (T1D) and type 2 (T2D) diabetes, and in subjects without diabetes (controls) in a cross-sectional study. All subjects underwent a lipidomic analysis using ultra-high performance liquid chromatography–electrospray ionization tandem mass spectrometry, carotid ultrasound (mode B) to assess SCA, and clinical assessment. Multiple linear regression models were used to assess the association between features and the presence and burden of SCA in subjects with T1D, T2D, and controls separately. Additionally, multiple linear regression models with interaction terms were employed to determine features significantly associated with SCA within risk groups, including smoking habit, hypertension, dyslipidaemia, antiplatelet use and sex. Depending on the population under study, different confounding factors were considered and adjusted for, including sample origin, sex, age, hypertension, dyslipidaemia, body mass index, waist circumference, glycated haemoglobin, glucose levels, smoking habit, diabetes duration, antiplatelet use, and alanine aminotransferase levels.

**Results:**

A total of 513 subjects (151 T1D, 155 T2D, and 207 non-diabetic control) were included, in whom the percentage with SCA was 48.3%, 49.7%, and 46.9%, respectively. A total of 27 unique lipid species were associated with SCA in subjects with T2D, in former/current smokers with T2D, and in individuals with T2D without dyslipidaemia. Phosphatidylcholines and diacylglycerols were the main SCA-associated lipidic classes. Ten different species of phosphatidylcholines were up-regulated, while 4 phosphatidylcholines containing polyunsaturated fatty acids were down-regulated. One diacylglycerol was down-regulated, while the other 3 were positively associated with SCA in individuals with T2D without dyslipidaemia. We discovered several features significantly associated with SCA in individuals with T1D, but only one sterol could be partially annotated.

**Conclusions:**

We revealed a significant disruption of lipid metabolism associated with SCA in subjects with T2D, and a larger SCA-associated disruption in former/current smokers with T2D and individuals with T2D who do not undergo lipid-lowering treatment.

**Graphical abstract:**

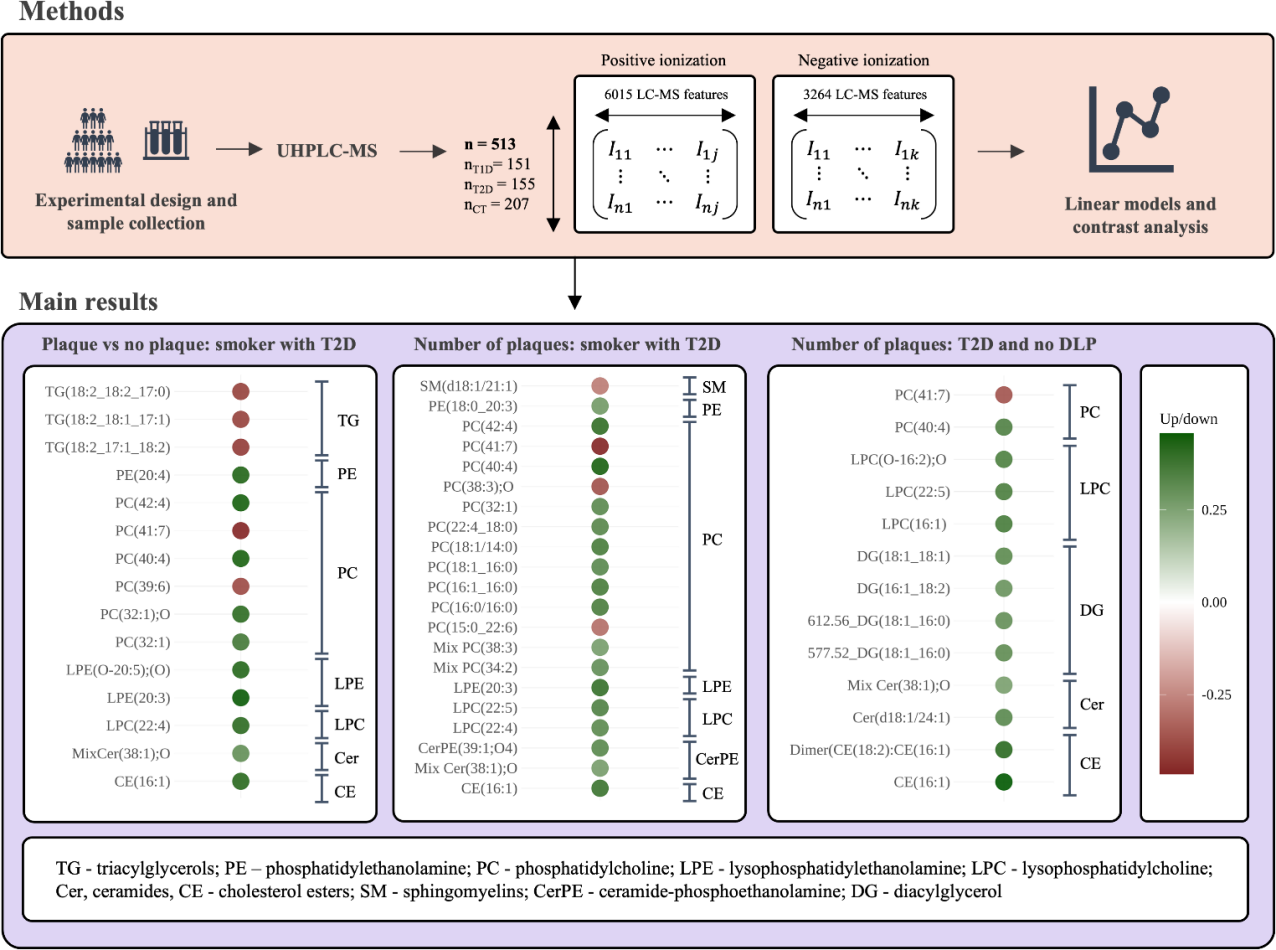

**Supplementary Information:**

The online version contains supplementary material available at 10.1186/s12933-025-02701-z.

## Research Insights

- Atherosclerosis progression is accelerated in individuals with diabetes. However, the underlying mechanisms behind this are not fully understood.
- Through an untargeted lipidomics approach, we investigated lipids associated with subclinical carotid atherosclerosis (SCA) in subjects with type 1 diabetes (T1D), type 2 diabetes (T2D) and those without diabetes.
- We found 27 lipids associated with SCA in individuals with T2D, including former/current smokers with T2D and those with T2D who do not take lipid-lowering treatment.
- All lipids associated with SCA except one were annotated exclusively in subjects with T2D, potentially explaining the poorer outcome of atherosclerosis in T2D. Similarly, we found a more disrupted lipid metabolism in former/current smokers with T2D and subjects with T2D who do not take lipid-lowering treatment.

**Supplementary Information:**

The online version contains supplementary material available at 10.1186/s12933-025-02701-z.

## Introduction

Atherosclerosis is the leading cause of cardiovascular (CV) disease worldwide, which in turn is the leading cause of death [1]. Diabetes is an important risk factor for CV disease. Both type 1 (T1D) and type 2 (T2D) diabetes are associated with accelerated atherosclerosis and a higher incidence of CV complications [2]. Although the risk of CV death is twofold higher in subjects with T2D [3], and 2 to 4 times greater in subjects with T1D [4], the underlying molecular mechanisms linking diabetes with accelerated atherosclerosis are not fully understood [2]. Furthermore, in a T2D population, the Framingham risk score has been shown to underestimate the CV risk [5], while the United Kingdom Prospective Diabetes Study score has been shown to overestimate the CV risk [6]. Moreover, subclinical carotid atherosclerosis (SCA) has a higher predictive power than that of classic CV risk factors and its inclusion in CV risk equations improves the performance of risk prediction [7].

Hyperglycaemia is a determining factor for the progression and severity of the atherogenic process. Intensive treatment to achieve near normoglycemia in T1D was shown to reduce the risk of any CV event by 42 percent [8]. In addition, atheroma plaques from subjects with diabetes have larger necrotic cores, and are more inflammatory and more vulnerable than similar size plaques from non-diabetic subjects [9], thereby contributing to an increase in the risk of future CV events [2, 9]. Higher levels of free fatty acids in coronary lesions in people with diabetes compared to those without diabetes have also been identified, which could potentially stimulate local plaque inflammation [10].

The relationship between smoking and the development of CV disease has been widely confirmed in epidemiologic studies [11]. Age, blood pressure, total cholesterol, HDL cholesterol, and body mass index have been established as sex-independent long-term predictors of an increase in intima media thickness, while in women only triglyceride levels are predictors, and in men only low physical activity and smoking are predictors [12], showing potential sex differences in the evolution of the disease. Moreover, in another work, we have shown how sex can shape lipid alterations in diabetes mellitus [13], demonstrating the importance of considering sex-specific differences in lipidomics.

Lipids are crucial for cell function and serve a great variety of functions [14]. Disorders in cell lipid composition have been related to atherosclerosis. Nonetheless, conventional lipid biomarkers (total cholesterol, high-density lipoprotein (HDL), low-density lipoprotein (LDL) and triglycerides (TG)) may not reflect the complex alteration of lipid metabolism in diabetes driving CV risk. Lipidomics provides a powerful novel platform for the discovery of new lipid biomarkers associated with CV disease [15]. Lipid species and classes have been found to be associated with T1D [13, 16] and T2D [13, 17], coronary heart disease [18], acute or stable arterial disease and incident CV events [19], hence, lipids could provide useful biomarkers to diagnose SCA.

In the present study, we performed a lipidomic analysis using Ultra High-Performance Liquid Chromatography–Mass Spectrometry (UHPLC-ESI–MS/MS) to examine specific lipid species associated with SCA presence and burden in a population of individuals with T1D, T2D, and without diabetes. Furthermore, using contrast analysis, we uncovered risk-specific lipidic patterns associated with SCA, such as smoking habit, hypertension, dyslipidaemia (DLP), and sex.

## Methods

### Participants

This cross-sectional study included 536 participants, 156 with T1D, 159 with T2D, and 221 without diabetes and matched by sex and body mass index (BMI). They were selected from the University Hospitals Arnau de Vilanova (Lleida, Spain), Germans Trias i Pujol (Badalona, Spain), Clinic (Barcelona, Spain), and the Primary Care Center Mollerussa (Lleida, Spain) from previous studies [20–23] (Additional File 1—Figure S1). The inclusion criteria for all groups were an age range of 20–85 years, absence of established chronic kidney disease (defined as calculated glomerular filtration rate < 60 mL/min and/or urine albumin/creatinine ratio > 299 mg/g), and absence of known clinical cardiovascular events or associated revascularization procedures, including coronary heart disease, cerebrovascular disease, or peripheral vascular disease (including the diagnosis of diabetic foot disease).

Age, sex, tobacco exposure, and pharmacological treatment were recorded. Subjects were considered to have hypertension or dyslipidaemia if they were under anti-hypertensive or lipid-lowering treatment, respectively. Diabetes duration was extracted from the medical records. Anthropometric data, weight, height, waist circumference, and blood pressure were obtained using standard methods. The standard biochemical analysis included glucose and glycated haemoglobin (HbA1c), lipid profile, and estimated glomerular filtration rate calculated according to the Chronic Kidney Disease Epidemiology Collaboration equation [24].

Blood samples were collected in the fasting state, and blood tests were performed using standard laboratory methods [21]. Urine tests were performed in subjects with diabetes following standard laboratory methods. The American Diabetes Association criteria (HbAc1 < 6.5% or fasting plasma glucose < 126 mg/dL) [25] was used to classify subjects without diabetes. Blood samples for the lipidomic analyses were collected in the fasting state with EDTA tubes, processed immediately after extraction, and stored at − 80 °C at the biobanks of the participant centres until determination.

From the 536 samples, 23 were discarded due to technical problems, thus 513 participants including 151 with T1D, 155 with T2D, and 207 without diabetes were used for the analysis.

### Ethics statement

Protocols of this study were approved by the local ethics committees of the University Hospital Germans Trias i Pujol (PI-15-147), which followed the Declaration of Helsinki. All participants provided written informed consent.

### Subclinical carotid atherosclerosis ascertainment

High-resolution B-mode carotid artery ultrasonography was performed using a LOGIQ® E9 (General Electric, Wauwatosa, WI 53226, USA) equipped with a 15-MHz linear array probe or a Sequoia 512 (Siemens, North Rhine, Westphalia, Germany) equipped with a 15-MHz linear array probe used to explore the common and internal carotid territories and the bifurcation from the left and right carotid arteries. A standardized protocol to evaluate the presence of carotid plaques by ultrasound has been previously described [21]. Briefly, the presence of carotid plaques was defined according to the Mannheim consensus as follows: a focal structure that encroaches into the arterial lumen of at least 0.5 mm or 50% of the surrounding carotid intima-media thickness value or demonstrates a thickness of 1.5 mm, as measured from the media-adventitia interference to the intima-lumen surface [26].

### Sample preparation

Samples were randomly assigned to one of 6 batches. The sample order within each batch was randomized before sample preparation to reduce the impact of technical factors, and then again prior to measurement of the lipid profile by UHPLC-ESI–MS/MS. All serum samples were thawed on ice, and 50 µL aliquots were taken from each sample to form a pooled quality control (QC) sample that represented all samples included in the study. The pooled QC was vortexed, further aliquoted (50 µL), and stored at − 80 °C until the analysis of each of the 6 batches of QC samples. Lipid extraction involved combining 50 µL of the biological sample or QC with 150 µL of isopropanol (LC–MS grade), vortexed for 20 s, and centrifuged at 22,000 g for 20 min at 4 °C. 120 µL of the supernatant was transferred to a low recovery vial and transferred to the LC sample manager at 4 °C.

### Ultra-high-performance liquid chromatography–mass spectrometry

Samples were kept at 4 °C and analysed using UHPLC–MS methods with a Dionex UltiMate 3000 Rapid Separation LC system (Thermo Fisher Scientific, MA, USA) coupled with a heated electrospray Q Exactive Focus mass spectrometer (Thermo Fisher Scientific, MA, USA). Non-polar extracts were analysed on a Hypersil GOLD column (100 × 2.1 mm, 1.9 µm; Thermo Fisher Scientific, MA, USA).

Mobile phase A was a solution containing 10 mM ammonium formate and 0.1% formic acid in 60% acetonitrile/water, while mobile phase B was composed of 10 mM ammonium formate and 0.1% formic acid in 90% propan-2-ol and water. The flow rate was maintained at 0.40 mL/min with the following gradient profile: t = 0.0, 20% B; t = 0.5, 20% B; t = 8.5, 100% B; t = 9.5, 100% B; t = 11.5, 20% B; t = 14.0, 20% B. All changes occurred linearly with curve = 5. The column temperature was set to 55 °C, and the injection volume was 2μL. Data acquisition was conducted separately in positive and negative ionization modes within the mass range of 150–2000 m/z at a resolution of 70,000 (FWHM at m/z 200). Ion source parameters were: sheath gas = 50 arbitrary units, Aux gas = 13 arbitrary units, sweep gas 3 arbitrary units, spray voltage 3.5 kV (positive ion mode) and 3.1 kV (negative ion mode), Capillary temp = 263 °C, and Aux gas heater = 425 °C. Data dependent MS2 in ‘Discover mode’ was applied for the MS/MS spectral acquisition with the following configuration: resolution at 17,500 (FWHM at *m/z* 200), isolation width 3.0 m*/z*, stepped normalized collision energy at 20, 50 and 80%. Spectra were acquired at three mass ranges: 200–400 m*/z*, 400–700 m*/z* and 700–1500 m*/z* on the pooled QC samples. Thermo ExactiveTune (2.8 SP1 build 2806) software was used to control the instrument in both cases, with data acquired in profile mode.

At the beginning of each run, QC samples were obtained using both profile and dependent scan modes (i.e., 7 QCs MS1 only, 3 QCs with MS2). Then, every seventh injection included two QC samples at the end of the analytical batch. Preparation blank samples were analysed between the 5th and 6th QCs and at the conclusion of the analytical batch.

### Mass spectrometry raw data processing

The raw data obtained in each analytical batch underwent conversion from the instrument-specific format to the mzML file format utilizing the open-access ProteoWizard (version 3.0.11417) msconvert tool [27]. Deconvolution was conducted using the R package XCMS (version 1.46.0) [28], within the Galaxy workflow environment. To optimize XCMS peak picking parameters, Isotopologue Parameter Optimization (IPO—version 1.0.0) was employed [29]. Subsequently, a data matrix containing metabolite features (m/z-retention time pairs) versus samples was generated, with peak areas provided.

### Assessment of data quality and peak matrix filtering

The initial five quality control samples in each batch were utilized to stabilize the analytical system and consequently were excluded before data processing and analysis. Data matrices underwent correction for run-order drift in intensity for each lipid feature individually using the Quality Control-Robust Spline Correction algorithm [30] using the sbcms [31] R package. Principal Component Analysis was employed to detect and eliminate suspected outlier samples (identified through PCs 1 and 2, Hotelling T2 *p* < 0.05) within each batch, ensuring reliable correction. At the beginning and end of each run, blank samples were employed to eliminate features originating from non-biological sources. Any feature exhibiting an average intensity across QC samples less than 20 times the average intensity of the blank samples was subsequently excluded. Any sample with > 20% missing values was excluded from further analysis. Metabolite features with a relative standard deviation of the QC samples > 30% and present in less than 90% of the QC samples were deleted from the dataset. Features with a < 50% detection rate over all samples were also removed. No imputation was performed on missing values.

### Statistical analysis

For the clinical data of participants, continuous variables were summarised as mean (standard deviation), and categorical data as frequency (percentage) using the compareGroups R package [32].

Prior to the statistical analysis, Probabilistic Quotient Normalization [33], using the mean of the QC samples as a reference, was applied. The natural logarithm of the metabolite features was computed to reduce skewness and the data were scaled and centred.

Multiple linear regression models were used to assess the association of each metabolite feature with the presence of SCA and the number of atherosclerotic plaques (SCA burden) in subjects with T1D, T2D, and those without diabetes separately. All models were adjusted for sample origin and classical atherosclerotic and diabetic risk factors, including sex, age, hypertension, dyslipidaemia, BMI, waist circumference, smoking habit, HbA1c, and glucose. Diabetes duration was included in the T1D and T2D models, while antiplatelet and alanine aminotransferase levels, were considered in the T1D model due to their significant association with SCA in this population. Total triglycerides were excluded to prevent masking relevant lipid-related signals. False Discovery Rate (FDR) was controlled using the R package qvalue [34] (q < 0.05).

In addition, a contrast analysis incorporating interaction terms was implemented to determine lipids significantly associated with presence and burden of SCA within risk groups, defined by five categorical variables (smoking habit, hypertension, dyslipidaemia and antiplatelet use) significantly associated with SCA in our study population, as well as sex.

Individuals with any type of diabetes were analysed together. Models were adjusted for sample origin, sex, age, hypertension, dyslipidaemia, BMI, waist circumference, smoking habit, HbA1c, and glucose. Diabetes duration was included when analysing individuals with diabetes, as well as antiplatelet use and alanine aminotransferase levels. Again, total TG were excluded to prevent masking relevant lipid-related signals.

FDR was controlled using the R package q-value. In the Methods section of the Supporting Information, a detailed explanation of the steps implemented in the contrast analysis is provided, as well as a detailed description of the analyses performed in Table S1 (Additional File 1).

Significant features containing tandem mass spectrometry (MS/MS) data were manually annotated.

## Results

### Clinical and biological characteristics

Clinical and anthropometrical characteristics of the study groups according to the presence of SCA are shown in Table 1. Briefly, subjects with SCA were older, and had high systolic blood pressure in the three groups (T1D, T2D, and non-diabetic controls). Further, subjects with T1D and SCA exhibited higher levels of triglycerides and alanine aminotransferase, and lower levels of HDL cholesterol than subjects with T1D without SCA. Moreover, higher proportions had tobacco exposure and had received antiplatelet treatment than their counterparts without SCA.

**Table 1:** Clinical characteristics of individuals with type 1 diabetes (T1D), type 2 diabetes (T2D), and without diabetes (Control) by the presence of subclinical carotid atherosclerosis

	Control			T1D			T2D		
	No plaque	Plaque	*P*-value	No plaque	Plaque	*P*-value	No plaque	Plaque	*P*-value
	N = 110	N = 97		N = 78	N = 73		N = 78	N = 77	
Sex (Men)	56 (50.9%)	58 (59.8%)	0.253	38 (48.7%)	42 (57.5%)	0.357	40 (51.3%)	46 (59.7%)	0.369
Age (Years)	52.4 (11.9)	57.4 (11.8)	**0.003**	44.8 (6.78)	52.8 (9.56)	** < 0.001**	56.5 (9.05)	62.1 (8.52)	** < 0.001**
BMI (kg/m^2^)	26.3 (3.88)	27.0 (4.05)	0.203	25.7 (4.09)	26.6 (3.88)	0.2	31.5 (5.58)	31.1 (5.37)	0.645
Waist (cm)	95.1 (11.9)	97.5 (11.4)	0.145	89.0 (11.7)	91.9 (12.4)	0.146	105 (12.3)	105 (13.4)	0.901
Former/Current Smoker	47 (42.7%)	52 (53.6%)	0.154	32 (41.0%)	49 (67.1%)	**0.002**	29 (37.2%)	38 (49.4%)	0.172
sBP (mmHg)	122 (14.9)	128 (14.8)	**0.004**	127 (17.3)	134 (17.2)	**0.011**	134 (18.7)	140 (17.0)	**0.03**
dBP (mmHg)	77.1 (9.46)	78.9 (9.32)	0.184	74.5 (10.1)	73.3 (10.7)	0.49	79.9 (11.3)	79.1 (9.95)	0.664
Hypertension (Yes)	14 (12.7%)	35 (36.1%)	** < 0.001**	10 (12.8%)	42 (57.5%)	** < 0.001**	37 (47.4%)	52 (67.5%)	**0.018**
Dyslipidaemia (Yes)	30 (27.3%)	35 (36.1%)	0.225	30 (38.5%)	53 (72.6%)	** < 0.001**	38 (48.7%)	42 (54.5%)	0.572
Antiplatelet (Yes)	–	–	–	17 (21.8%)	35 (47.9%)	**0.001**	19 (24.4%)	24 (31.2%)	0.443
HbA1c (%)	5.45 (0.33)	5.49 (0.40)	0.382	7.53 (0.80)	7.65 (1.00)	0.413	7.78 (1.57)	7.65 (1.79)	0.625
HbA1c IFCC	36.1 (3.76)	36.6 (4.47)	0.428	58.8 (8.71)	60.2 (10.9)	0.406	61.5 (17.1)	60.1 (19.6)	0.627
Triglycerides (mg/dL)	113 (53.0)	109 (50.1)	0.575	68.5 (28.3)	86.4 (54.8)	**0.014**	136 (80.1)	149 (84.6)	0.303
Total cholesterol (mg/dL)	205 (35.4)	210 (32.3)	0.298	184 (26.4)	178 (31.8)	0.233	185 (38.7)	187 (44.2)	0.777
HDL cholesterol (mg/dL)	57.7 (13.0)	57.9 (14.5)	0.907	66.2 (17.1)	61.5 (15.4)	0.075	49.1 (13.1)	49.2 (13.1)	0.979
LDL cholesterol (mg/dL)	125 (30.1)	131 (29.6)	0.185	104 (20.8)	101 (27.8)	0.461	110 (33.3)	109 (36.4)	0.86
DM duration (years)	–	–	–	22.8 (9.77)	24.5 (11.7)	0.331	6.10 (7.05)	6.68 (8.14)	0.637
Retinopathy (Yes)	–	–	–	32 (41.6%)	41 (56.9%)	0.087	20 (29.4%)	28 (39.4%)	0.287
ALT	22.2 (14.8)	21.5 (19.3)	0.792	18.8 (8.86)	22.8 (10.5)	**0.012**	32.6 (23.8)	28.9 (14.1)	0.247

Plaque and No plaque columns indicate the individuals with and without atherosclerotic plaques, respectively. The P-value column shows the significance of the association between each clinical variable and the presence of plaque. Statistically significant values are highlighted in bold. BMI, body mass index; sBP, systolic blood pressure; dBP, diastolic blood pressure; HbA1c, glycated haemoglobin; HDL, high-density lipoprotein; LDL, low-density lipoprotein; DM, diabetes mellitus; ALT, alanine aminotransferase levels

### Lipids associated with subclinical carotid atherosclerosis presence and burden

Untargeted LC–MS was performed using 513 serum samples (151 T1D, 155 T2D, and 207 controls). The lipidomic study detected 6015 and 3264 LC–MS features in positive and negative acquisition modes. From which 980 were annotated with LipidSearch in positive acquisition mode and 208 in negative mode. After filtering, 5397 and 3098 features remained in positive and negative modes, respectively.

Tables S2 and S3 summarise the relative abundance of lipids annotated using LipidSearch for positive and negative ionization modes, respectively. The mean (standard deviation) lipid abundances are provided for the overall population, as well as for individuals without diabetes, and those with T1D and T2D.

From the remaining features, 68 were significantly associated with SCA burden in subjects with T1D, 26 with SCA presence in subjects with T2D, and 2 with SCA burden in subjects without diabetes.

Regarding the interaction analyses, in smokers with T1D and T2D, 20 and 181 unique features, respectively, were significantly associated with the presence and burden of SCA. In subjects with T1D without hypertension, 8 features were significantly associated with SCA, while only 1 was significant in those with hypertension. In subjects without dyslipidaemia, 16 and 55 unique features were significantly associated with the presence or burden of SCA in T1D and T2D, respectively, whereas in individuals with dyslipidaemia, only 1 feature was significantly associated with SCA in T1D. In subjects with T1D who did not take antiplatelet agents, 33 features were significantly associated with SCA, while only 1 feature was significant in those taking antiplatelet drugs. Regarding sex, 1 and 6 SCA-associated features were found in men with T1D and T2D, respectively, while 34 and 4 were found in women with T1D and T2D, respectively. In subjects without diabetes, 3 features were significantly associated with SCA in smokers and 4 in subjects with hypertension.

A total of 27 unique lipid species belonging to glycerophospholipid, glycerolipid, sphingolipid and sterol lipid families were annotated from the SCA-associated features (Tables 2 and 3). The most altered lipidic family was glycerophospholipids, representing 58.97% of the significant lipid species. From these, phosphatidylcholines (PC) were the main class, accounting for 65.22%, followed by lysophosphatidylcholines (LPC, 17.39%), phosphatidylethanolamines (PE, 8.70%) and lysophosphatidylethanolamines (LPE, 8.70%). Glycerolipids represented 20.51% of the significant lipids, diacylglycerols (DG) 62.5%, and triacylglycerols (TG) 37.5%. Sphingolipids represented 12.82%, from which ceramides (Cer) represented 60% and sphingomyelins (SM) and ceramide-phosphoethanolamine (CerPE) represented both 20%. Sterol lipids (7.69%) were the least represented lipidic family, with two cholesterol esters (CE) and one sterol lipid with chemical formula C_29_H_50_O.

**Table 2:** Lipids significantly associated with SCA presence. All lipids were manually annotated using MS/MS data

mz	rt	Lipids	q-value	Beta	n	Analysis
1289.1869	597.06	Dimer(CE(18:2):CE(16:1)) + NH4	0.037; 0.048	0.84 [0.45,1.23]; 0.68 [0.34,1.03]	285; 144	T2D no DLP; T2D
502.2925	107.44	LPE(O-20:5);(O) + H	0.036	0.81 [0.39,1.24]	285	Smokers with T2D
504.3084	130.8	LPE(20:3) + H	0.033	0.88 [0.44,1.31]	282	Smokers with T2D
524.2746	107.19	PE(20:4) + Na	0.036	0.82 [0.38,1.26]	285	Smokers with T2D
572.3709	153.57	LPC(22:4) + H	0.033	0.79 [0.42,1.17]	285	Smokers with T2D
578.5869	517.93	Mix Cer(38:1); O − main Cer(m18:1/20:0) + H	0.042	0.59 [0.27,0.92]	264	Smokers with T2D
601.5185	601.59	DG(18:1_18:2) − H_2_O + H	0.048	− 0.69 [− 1.06,− 0.32]	139	T2D
640.6023	596.54	CE(16:1) + NH4	0.036; 0.044; 0.037	0.8 [0.38,1.22]; 0.74 [0.38,1.1]; 0.92 [0.51,1.33]	244; 135; 244	Smokers with T2D; T2D; T2D no DLP
732.5531	459.75	PC(32:1) + H	0.036	0.72 [0.33,1.1]	285	Smokers with T2D
748.5472	427.64	PC(32:1);O + H	0.036	0.79 [0.39,1.19]	285	Smokers with T2D
820.5834	461.35	PC(39:6) + H	0.046	− 0.75 [− 1.16,− 0.33]	285	Smokers with T2D
838.6313	499.04	PC(40:4) + H	0.033	0.84 [0.43,1.26]	285	Smokers with T2D
846.5993	428.71	PC(41:7) + H	0.036	− 0.89 [− 1.34,− 0.44]	259	Smokers with T2D
866.6634	514.92	PC(42:4) + H	0.033	0.85 [0.43,1.27]	279	Smokers with T2D
884.7695	587.96	TG(18:2_17:1_18:2) + NH4	0.038	− 0.8 [− 1.24,− 0.37]	285	Smokers with T2D
886.7851	597.06	TG(18:2_18:2_17:0) + NH4	0.048; 0.048	− 0.67 [− 1.02,− 0.31]; − 0.78 [− 1.22,− 0.34]	144; 285	T2D; Smokers with T2D
886.7851	597.06	TG(18:2_18:1_17:1) + NH4	0.048; 0.048	− 0.67 [− 1.02,− 0.31]; − 0.78 [− 1.22,− 0.34]	144; 285	T2D; Smokers with T2D

m/z, mass-to-charge ratio value; rt, retention time; q-value, list of corrected p-values for each analysis where the lipid is considered to be significant, the p-values are obtained from the student’s t-tests performed in linear models; Beta, list of linear regressors for each significant analysis with their 95% confidence interval between brackets; n, number of observations in each model; Analysis, analysis where the lipid is considered significant (subjects with T2D—T2D, subjects with T2D without dyslipidemia—T2D no DLP, and smokers with T2D—Smokers with T2D). All lipids were acquired in positive acquisition mode

**Table 3:** Lipids significantly associated with SCA burden. All lipids were manually annotated using MS/MS data

mz	rt	Lipids	q-value	Beta	n	Analysis
1289.1869	597.06	Dimer(CE(18:2):CE(16:1)) + NH4	0.021	0.4 [0.22,0.57]	285	T2D no DLP
494.324	88.44	LPC(16:1) + H	0.047	0.35 [0.16,0.54]	281	T2D no DLP
494.3241	98.07	LPC(O-16:2);O + H	0.048	0.34 [0.15,0.52]	285	T2D no DLP
504.3084	130.8	LPE(20:3) + H	0.03	0.43 [0.22,0.64]	282	Smokers with T2D
570.3558	131.32	LPC(22:5) + H	0.046; 0.047	0.38 [0.17,0.58]; 0.34 [0.15,0.53]	245	Smokers with T2D; T2D no DLP
572.3709	153.57	LPC(22:4) + H	0.041	0.35 [0.17,0.54]	285	Smokers with T2D
577.5182	514.69	DG(18:1_16:0)-H_2_O + H	0.047	0.3 [0.14,0.47]	285	T2D no DLP
578.5869	517.93	Mix Cer(38:1);O − main Cer(m18:1/20:0) + H	0.049; 0.047	0.25 [0.11,0.39]; 0.29 [0.13,0.45]	264	T2D no DLP; Smokers with T2D
608.5246	479.45	DG(16:1_18:2_0:0) + NH4	0.047	0.28 [0.13,0.44]	285	T2D no DLP
612.556	515.31	DG(18:1_16:0_0:0) + NH4	0.047	0.29 [0.13,0.46]	285	T2D no DLP
621.5448	516.82	DG(18:1_18:1) + H	0.047	0.31 [0.14,0.47]	270	T2D no DLP
640.6023	596.54	CE(16:1) + NH4	0.03; 0.013	0.42 [0.21,0.63]; 0.44 [0.26,0.63]	244	Smokers with T2D; T2D no DLP
648.6288	539.32	Cer(d18:1/24:1) + H	0.047	0.31 [0.14,0.48]	285	T2D no DLP
732.5531	459.75	PC(32:1) + H	0.042	0.35 [0.17,0.54]	285	Smokers with T2D
828.6096	480.61	PC(38:3);O + H	0.041	− 0.41 [− 0.62,− 0.19]	285	Smokers with T2D
838.6313	499.04	PC(40:4) + H	0.01; 0.047	0.48 [0.28,0.68]; 0.33 [0.15,0.51]	285	Smokers with T2D; T2D no DLP
846.5993	428.71	PC(41:7) + H	0.01; 0.047	− 0.5 [− 0.72,− 0.28]; − 0.35 [− 0.54,− 0.16]	259	Smokers with T2D; T2D no DLP
866.6634	514.92	PC(42:4) + H	0.022	0.44 [0.23,0.64]	279	Smokers with T2D
397.3826	607.15	Sterol lipid − C_29_H_50_O	0.044	− 0.37 [− 0.56, − 0.17]	142	T1D
768.556	496.59	PE(18:0_20:3)-H	0.048	0.3 [0.12,0.49]	269	Smokers with T2D
776.5454	461.5	Mix PC (32:1) − PC(16:1_16:0) + HCOO– & PC(18:1/14:0) + HCOO–	0.018	0.39 [0.2,0.59]	269	Smokers with T2D
778.5611	482.89	PC(16:0/16:0) + HCOO–	0.032	0.39 [0.17,0.6]	269	Smokers with T2D
802.5609	465.5	Mix PC(34:2) − PC(18:2_16:0) + HCOO– & PC(18:1_16:1) + HCOO–	0.049	0.34 [0.12,0.55]	269	Smokers with T2D
804.5765	484.96	PC(18:1_16:0) + HCOO–	0.048	0.36 [0.14,0.57]	269	Smokers with T2D
807.586	484.96	CerPE(39:1;O4) + HCOO-	0.048	0.36 [0.14,0.57]	269	Smokers with T2D
815.6307	497.13	SM(d18:1/21:1) + HCOO–	0.049	− 0.3 [− 0.49,− 0.11]	269	Smokers with T2D
836.5458	439.16	PC(15:0_22:6) + HCOO-	0.05	− 0.33 [− 0.55,− 0.12]	267	Smokers with T2D
856.608	494.63	Mix PC(38:3) − main PC(18:2_20:1) + HCOO– & PC(18:1_20:2) + HCOO–	0.049	0.29 [0.11,0.47]	269	Smokers with T2D
882.624	500.35	PC(22:4_18:0) + HCOO–	0.049	0.36 [0.14,0.58]	269	Smokers with T2D

m/z, mass-to-charge ratio value; rt, retention time; q-value, list of corrected p-values for each analysis where the lipid is considered to be significant, the p-values are obtained from the student’s t-tests performed in linear models; Beta, list of linear regressors for each significant analysis with their 95% confidence interval between brackets; n, number of observations in each model; Analysis, analysis where the lipid is considered significant (subjects with T1D–T1D, subjects with T2D–T2D, subjects with T2D without dyslipidemia—T2D no DLP, and smokers with T2D—Smokers with T2D). All lipids were acquired in positive acquisition mode except the ones with the adduct [M + HCOO–]

When analysing SCA presence and SCA burden, the general trends observed in lipid class associations remained consistent in individuals with T2D. PEs, PCs, LPEs, LPCs, ceramides and CEs were positively associated with SCA in multiple sub-analyses.

Conversely, polyunsaturated TGs and PCs enriched in highly polyunsaturated fatty acids, specifically PC(41:7), PC(39:6) and PC(15:0_22:6), were negatively associated with SCA across several groups. In individuals with T2D, DG(18:1_18:2) was negatively associated with the presence of plaque, however, a set of three DGs were significantly increased in individuals with SCA and without dyslipidaemia. In smokers with T2D, SM(d18:1/22:6) was negatively associated with SCA burden.

A dimer of CE(16:1) and CE(18:2), as well as CE(16:1) alone, was significantly increased in individuals with T2D and in individuals with T2D and without dyslipidaemia.

In individuals with T1D, several features were significantly associated with SCA burden across the analysed sub-groups; however, only one could be partially annotated. A sterol with the formula C_29_H_50_O obtained a q-value of 0.047 and a linear regressor of − 0.37.

**Fig. 1:**
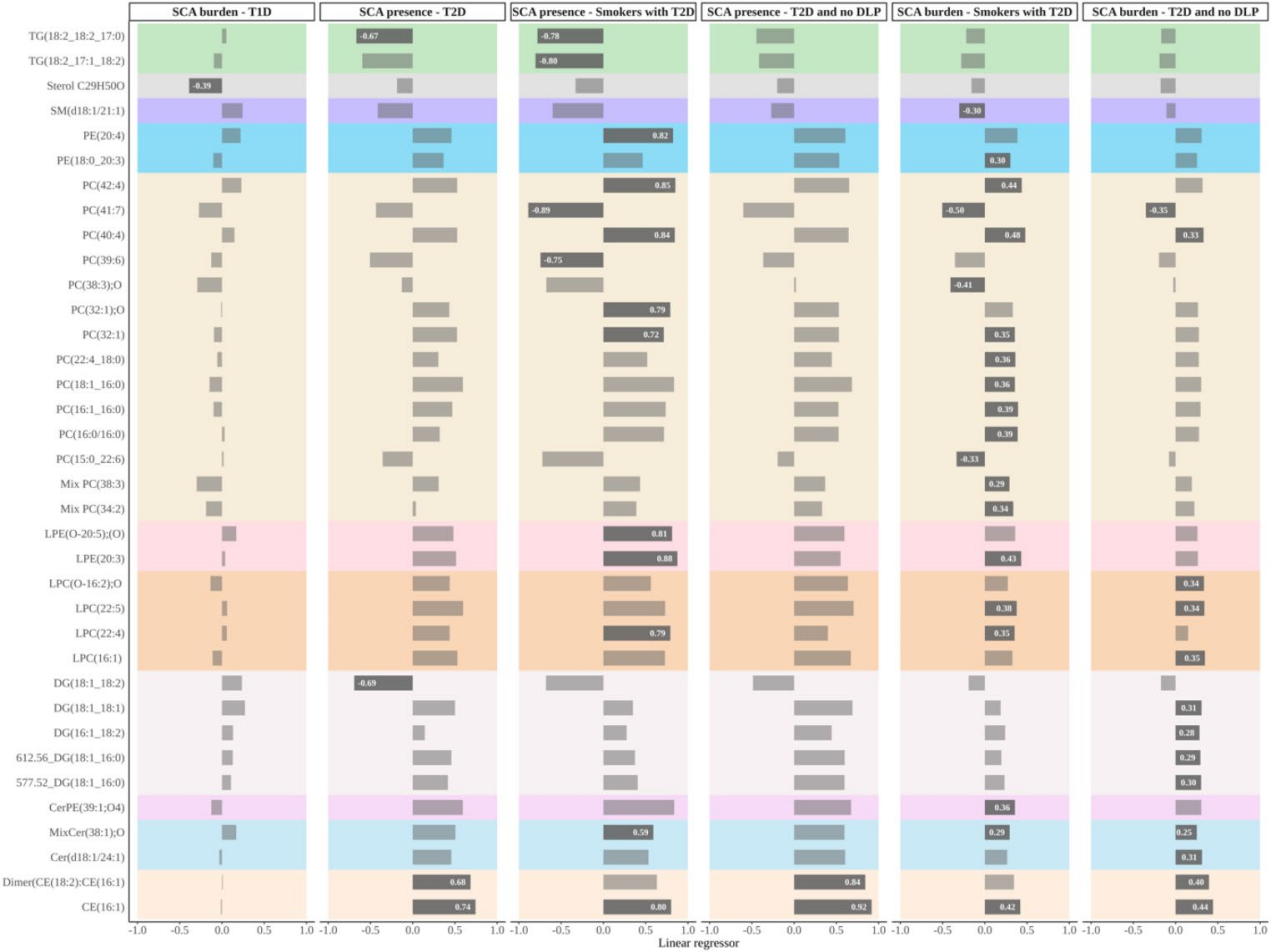
Bar plot of the linear regressors of lipids associated with SCA in six analyses. From left to right, the columns show lipids associated with: the burden of SCA in subjects with T1D, the presence of SCA in subjects with T2D, the presence of SCA in smokers with T2D, the presence of SCA in subjects with T2D and without dyslipidaemia, the burden of SCA in smokers with T2D and the burden of SCA in subjects with T2D and without dyslipidaemia. Colours differentiate lipid classes. Darker bars with the linear regressor value in white indicate significance. TG, triacylglycerol; SM, sphingomyelin; PE, phosphatidylethanolamine, PC, phosphatidylcholine; LPE, lysophosphatidylethanolamine; LPC, lysophosphatidylcholine, DG, diacylglycerol; CerPE, ceramide-phosphoethanolamine; Cer, ceramide; CE, cholesterol ester. Mix indicates an LC–MS feature that includes multiple lipid species with the same sum composition notation. For example, Mix PC(38:3) represents PC species containing a total of 38 carbon atoms and 3 double bonds

Detailed data supporting these findings is provided in Tables 2 and 3 and Fig. 1.

The lipids significantly associated with the presence/burden of SCA in subjects with T1D, T2D, in former/current smokers with T2D and individuals with T2D and without dyslipidaemia are shown in Fig. 1.

**Fig. 2:**
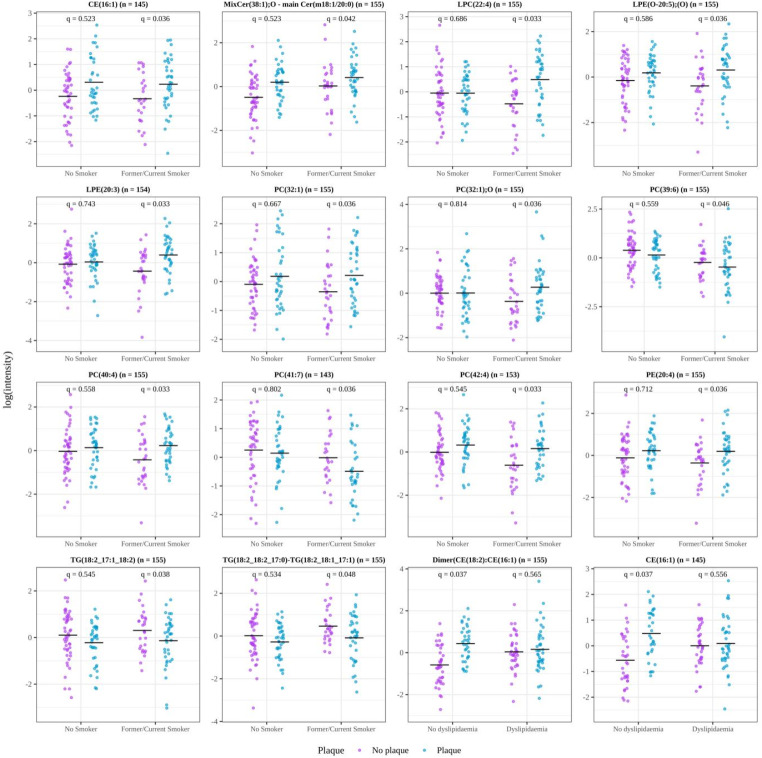
Boxplots of the scaled and centred log-transformed intensity of each lipid significantly associated with the presence of SCA in smokers with T2D and in individuals with T2D without dyslipidaemia. All lipids shown correspond to LC–MS features acquired in positive ionization mode. The first 14 plots correspond to the smoking habit comparison, while the last 2 represent the dyslipidaemia comparison. Each plot title displays the lipid name and the number of observations. The x-axis labels indicate the sub-group of individuals analysed. The q-values for each comparison are displayed at the top of the plots. CE, cholesterol esters; Cer, ceramides; LPC, lysophosphatidylcholine; LPE, lysophosphatidylethanolamine; PC, phosphatidylcholine; PE, phosphatidylethanolamine; TG, triacylglycerols. Mix indicates an LC–MS feature that includes multiple lipid species with the same sum composition notation

Figure 2 shows a boxplot of the log-transformed intensity of each lipid significantly associated with SCA presence in former/current smokers with T2D and individuals with T2D and without dyslipidaemia.

## Discussion

We measured serum lipid species in T1D, T2D, and subjects without diabetes with and without SCA. We found evidence that several lipids are related to the presence and burden of SCA in T2D, especially in former/current smokers and individuals without lipid-lowering treatment (without dyslipidaemia). To the best of our knowledge, this is the first lipidome–wide association study focused on determining associations between lipid species and SCA through an untargeted lipidomic serum profiling in a population composed of T1D, T2D, and subjects without diabetes and determining risk factor-specific differences.

Our work revealed lipid metabolism disruption in subjects with SCA. The analyses showed LC–MS features associated with the presence and burden of SCA in individuals with T1D and without diabetes. However, the majority of features that could be annotated were associated with SCA in subjects with T2D. These results might be linked with the accelerated progression of atherosclerotic lesions in diabetes, as well as the presence of larger necrotic cores in advanced atherosclerotic lesions of subjects with diabetes compared to subjects without diabetes [2].

In individuals with T1D, several features were significantly associated with SCA, but only one could be partially annotated as a sterol lipid with the formula C_29_H_50_O. This lipid was significantly reduced in individuals with SCA. However, due to the partial annotation, no conclusions can be drawn regarding its role in SCA and T1D. The lack of annotated lipids in the analysis of individuals with T1D may suggest a more favourable lipidomic profile associated with SCA in T1D compared to T2D, at least within our population. However, this contrasts with previous literature, as mentioned earlier [4]. Another possible explanation is the greater heterogeneity of our T1D population, as shown in Table 1. This could lead to increased variability in the confounding factors included in the models, amplifying their effects and potentially masking lipid signals associated with SCA.

Another finding of this study is the significant disruption of lipid metabolism associated with SCA in former/current smokers compared with non-smokers and in individuals without dyslipidaemia, as evidenced by the higher number of significant LC–MS features in these sub-groups in T1D and T2D (Additional File 1—Table S4). The lipids reported in this work may have biological implications in the underlying mechanisms behind the association between SCA and smoking habit [35, 36]. On the other hand, T2D is associated with dyslipidaemia, especially atherogenic dyslipidaemia. It is well known that these lipoprotein abnormalities are leading to an increased risk of CV disease [37]. Therefore, although in the current study we cannot relate the lipidomic alterations to specific lipoprotein abnormalities in T2D subjects, it is conceivable that, at least in part, some lipidomic features found in our study are originally part of the lipoprotein profile of T2D. In relation to this, lipid-lowering treatment has been shown to improve coronary artery disease [37]. In this study, dyslipidaemia is defined as the use of lipid-lowering treatment. Therefore, the lipid alterations associated with SCA in individuals without dyslipidaemia, compared to those with dyslipidaemia, may explain the beneficial effect of these treatments on SCA.

On the other hand, regarding sex-specific differences, Table S4 (Additional File 1) shows a more significant disruption of lipid metabolism associated with SCA in women with T1D than in their male counterparts. In contrast, in subjects with T2D, 5 features were significantly associated with SCA only in men. Although none of these features could be annotated, PC(42:4) and TG(18:2_17:1_18:2) had q-values of 0.051 in the analysis for men, while their q-values were 0.47 and 0.52, respectively, in women. PC(42:4) was positively associated with SCA in men with T2D, whereas TG(18:2_17:1_18:2) showed a negative association. The trends observed for these lipids were consistent across other subgroups, which may suggest a worse lipidomic profile associates to SCA in men with T2D compared to women with T2D. The role of sex in lipid metabolism and diabetes has been already shown in our previous work [13]. Further, it should be pointed out that other studies have demonstrated differences between men and women, with less plaque rupture and necrotic core in atherosclerosis in the latter [38]. The lipids identified in this study may help explain some of these phenomena.

### Glycerophospholipids

Glycerophospholipids were the main altered lipidic family, with a high representation of phosphatidylcholines (PC), of which fourteen were associated with SCA in at least one of the analyses performed. We have found a general SCA-associated increase in PC species. It has been shown that increasing PC synthesis constitutes an adaptive response to the accumulation of free cholesterol in macrophages in atherosclerotic lesions. This response prevents a toxic free-cholesterol:phospholipid ratio, delaying macrophage death [39]. Additionally, macrophage necrosis increases the risk of plaque rupture, potentially leading to thrombosis-related cardiovascular events [40]. Since we included subjects without previous CV events, it is tempting to speculate that the characteristic metabolic patterns revealed in this study are indicative of the initial stages of atherosclerosis. In agreement with our findings, PC(32:0) has been previously associated with an increased risk of cardiovascular events [41] and coronary artery disease (CAD) mortality [42], while PC(32:1), with total mortality [42] and CV disease risk [19]. Moreover, PC(40:4) and PC(32:1) have been previously reported to be associated with smoking habits in men and women, respectively, with the authors discussing a possible link between smoking, these PCs, and the pathogenesis and prognosis of CV disease [43]. PC(34:1) and PC(34:2) have also been positively associated with CAD and total mortality [42], which agrees with our findings. On the other hand, several highly polyunsaturated PCs, such as PC(40:7) or PC(38:3), have been negatively associated with total mortality and CAD [42], which concurs with our findings since PC(41:7), PC(39:6), PC(15:0_22:6) and PC(38:3);O have been linked to the absence of SCA. Additionally, PCs containing long-chain polyunsaturated fatty acids were consistently elevated in surviving heart failure patients. PC(37:6) was one of the PCs significantly associated with heart failure survival [44].

Lysophosphatidylcholines (LPC) are generally involved in several proatherogenic mechanisms, such as endothelial dysfunction or macrophage proliferation. We have shown a positive association of LPC(22:4) with SCA presence and burden and LPC(22:5) with SCA burden in former/current smokers with T2D and individuals with T2D and without dyslipidaemia. Specifically, LPC(22:4) contains adrenic acid, which aggravates inflammation, and it has been positively associated with the initial stages of the development of atherosclerotic lesions in WHHLMI rabbits [45]. Additionally, LPC(22:5) has been linked to age in ApoE^−/−^ mice and has been identified as a human atherosclerotic tissue marker [46]. We also found a positive association between LPC(O-16:2);O and LPC(16:1) and SCA in individuals with T2D who are not undergoing lipid-lowering treatment. A study demonstrated an increase in LPC(16:1) and other phospholipids in monocytes overexpressing long-chain fatty acyl-CoA synthetase 1 (ACSL1). ACSL1 is upregulated in both hyperlipidaemia and acute myocardial infarction. This research emphasized the role of ACSL1 in linking hyperlipidaemia to myocardial ischemia–reperfusion injury, particularly through phospholipids like LPC(16:1). They linked these findings with previous research showing that these phospholipids contribute to pathological processes associated with thrombosis [47]. Our findings align with these observations and may suggest a more favourable SCA-associated lipid profile in individuals receiving lipid-lowering treatment.

In the present study, two lysophosphatidylethanolamines (LPE) were positively associated with SCA in former/current smokers with T2D. LPE(20:3) was increased in mice developing atherosclerosis due to hepatic *Plpp3* deletion [48]. To our knowledge, LPE(O-20:5);O, has not been previously associated with atherosclerosis. However, LPE(O-20:5);O, together with PC(32:1);O and LPC(O-16:2);O are glycerophospholipids containing an hydroxy fatty acid. The increase of hydroxy fatty acids has been previously associated with LDL oxidation stage, age [49], and atherosclerosis [50], which could explain the increase of these lipids in the population under study. In addition, two phosphoethanolamines (PE), PE(20:4) and PE(18:0_20:3), were positively associated with the presence and burden of SCA, respectively, in former/current smokers with T2D. PE(38:3) has previously been found to be significantly increased in HDL particles of patients with acute coronary syndrome compared to those with stable coronary artery disease [51]; this is in line with our findings. However, as far as we know, this is the first study reporting a significant association between PE(20:4) and SCA or other cardiovascular-related conditions.

### Glycerolipids

Glycerolipids were also significantly modified, with diacylglycerols (DG) being the most altered class. We found DG(18:1_18:2) to be significantly reduced in subjects with SCA and T2D. Additionally, two polyunsaturated triacylglycerols (TG), TG(18:2_18:2_17:0) and TG(18:2_17:1_18:2), were negatively associated with the presence of SCA in subjects with T2D and smokers with T2D. Species of glycerolipids, such as the TGs and DGs containing 18:2 fatty acids, have been linked to a reduced risk of CV death [41]. Long-chain polyunsaturated fatty acids have also been previously associated with a protective effect in CV disease [42]. Furthermore, some studies have suggested that a reduction in Stearoyl Coenzyme Desaturase-1 (SCD-1) activity, which is responsible for the unsaturation of palmitate (16:0) and stearate (18:0), may contribute to atherosclerosis [42, 52]. This evidence could explain the observed reduction of TGs and DGs containing palmitoleate (16:1n-7), oleate (18:1n-9), and long polyunsaturated fatty acids in our study. However, we also found DG(18:1_18:1), DG(16:1_18:2), and DG(18:1_16:0) to be positively associated with SCA burden in individuals not undergoing lipid-lowering treatment, which could contradict the previous hypotheses. Therefore, further research is needed to clarify the role of DG species in SCA among individuals with T2D undergoing lipid-lowering treatment.

### Sphingolipids

Regarding sphingolipids, we found that one sphingomyelin (SM), SM(d18:1/21:1), was negatively associated with SCA burden in former/current smokers with T2D. Additionally, two ceramides (Cer) were positively associated with SCA across several groups, and one ceramide-phosphoethanolamine (CerPE) was positively associated with SCA burden specifically in smokers with T2D. Cer(m18:1/20:0) was significantly increased with SCA in smokers with T2D and in individuals with T2D and without dyslipidaemia, and Cer(d18:1/24:1) in individuals with T2D without dyslipidaemia. An increase in plasma Cer(m18:1/20:0) has been previously linked to epicardial adipose tissue volume, which has been described to be associated with coronary artery disease [53]. Moreover, plasma ceramides—specifically Cer(d18:1/24:1)—have been shown to predict high atherosclerotic burden in patients with ST-segment elevation myocardial infarction [54]. Moreover, a high ratio of Cer(d18:1/24:1) to Cer(d18:1/24:0) has been associated with the severity of coronary artery stenosis [55]. The association that we have found between Cer(d18:1/24:1) and SCA exclusively in individuals with T2D who are not receiving lipid-lowering treatment may reflect the beneficial effect of this therapy on the lipid profile. We have also found a positive association between SCA burden and CerPE(39:1;O4). To the best of our knowledge, this is the first study reporting a significant association between this lipid and SCA or other cardiovascular-related conditions.

### Sterol lipids

Our findings revealed two cholesterol esters (CE) positively associated with SCA. CE(16:1) was associated with SCA presence and burden in subjects with T2D and former/current smokers with T2D, and CE(18:2) was associated with SCA presence in subjects with T2D. Consistent with these results, CE(16:1) has been used as a biomarker to improve risk classification for CV disease [19] and has been associated with acute coronary syndrome [56] and, together with CE(18:2), with incident myocardial infarction [57].

This study has several strengths. First, it includes an appropriate sample size (n = 513) for statistical analysis. Secondly, the population comprises subjects without diabetes, with T1D and with T2D, enabling the evaluation of SCA-associated changes in metabolism in a diabetes-specific way. Moreover, the contrast analyses show differences due to tobacco exposure and the use of lipid-lowering treatment. Finally, a comprehensive set of variables to minimize confounding is used. However, it also has some limitations. First, SCA burden variable is not balanced, probably weakening the signal and increasing the rate of false negatives. Secondly, the observational nature of our study does not allow us to make causal inferences; thus, the biochemical mechanisms presented herein cannot be fully elucidated. Third, we acknowledge that annotation using MS/MS technology may introduce errors; therefore, further research is needed to confirm the annotated lipids. Finally, validation of the current findings in an independent cohort is very relevant to confirm the association of the annotated lipids with SCA.

## Conclusions

In conclusion, this is the first study to reveal specific lipids significantly associated with SCA in subjects with T1D, T2D, and without diabetes without known previous CV events, together with risk factors-specific lipid differences associated with SCA. Moreover, we have demonstrated greater SCA-related disruption in lipid metabolism in subjects with T2D compared to those with T1D or without diabetes, as well as more pronounced disruption in former or current smokers and individuals not undergoing lipid-lowering treatment. Our findings demonstrate the power of lipidomics to discover new biomarkers for preventive medicine in cardiovascular research. However, validating the annotated lipids found in this work in an independent cohort is required to confirm them as potential SCA biomarkers.

## Supplementary Information

Additional file 1

## Data Availability

The datasets used and/or analysed during the current study are available from the corresponding author on reasonable request.

## Abbreviations

SCA: Subclinical carotid atherosclerosis
T1D: Type 1 diabetes
T2D: Type 2 diabetes
UHPLC-ESI**–**MS/MS: Liquid chromatography–electrospray ionization tandem mass spectrometry
CV: Cardiovascular
HDL: High-density lipoprotein
LDL: High-density lipoprotein
TG: Triglycerides
LC**–**MS: Liquid chromatography–mass spectrometry
DLP: Dyslipidaemia
BMI: Body mass index
HbA1c: Glycated haemoglobin
QC: Quality control
FDR: False discovery rate
sBP: Systolic blood pressure
dBP: Diastolic blood pressure
DM: Diabetes mellitus
ALT: Alanine aminotransferase
MS/MS: Tandem mass spectrometry
PC: Phosphatidylcholines
LPC: Lysophosphatidylcholines
PE: Phosphatidylethanolamines
LPE: Lysophosphatidylethanolamines
DG: Diacylglycerols
TG: Triacylglycerols
Cer: Ceramides
SM: Sphingomyelins
CerPE: Ceramide-phosphoethanolamine
CE: Cholesterol esters
CAD: Coronary artery disease
