# Supplementary material for: Lipidomic analysis reveals metabolism alteration associated with subclinical carotid atherosclerosis in type 2 diabetes
Source: Cardiovasc Diabetol. 2025 Apr 2;24:152. doi: 10.1186/s12933-025-02701-z (PMC11967040; doi:10.1186/s12933-025-02701-z)
Supplement: Supplementary file 1 — Additional file 1 [file 12933_2025_2701_MOESM1_ESM.pdf]

# Detailed Methods - Lipidomic analysis reveals metabolism alteration associated with subclinical carotid atherosclerosis in type 2 diabetes

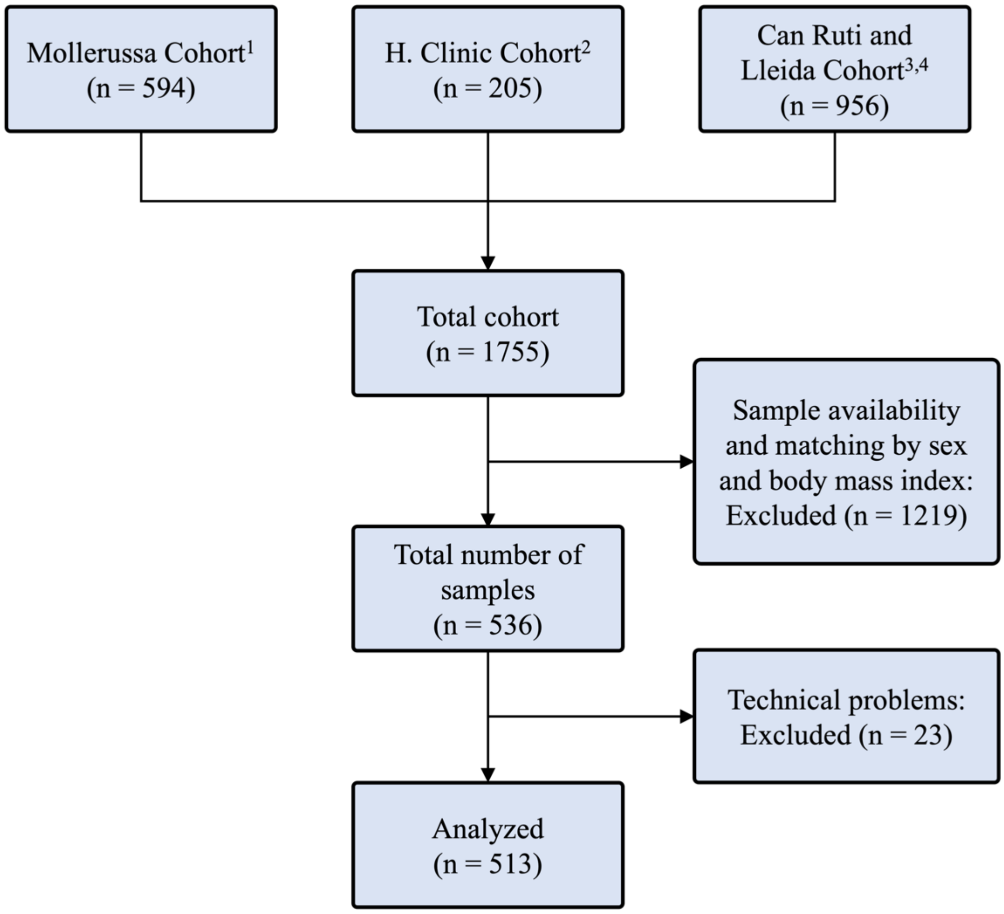


*Figure S1. Flow chart of the participants recruitment.*


(1) *Vilanova MB, Falguera M, Marsal JR, Rubinat E, Alcubierre N, Castelblanco E, Granado-Casas M, Miró N, Molló À, Mata-Cases M, Franch-Nadal J, Mauricio Di. Prevalence, clinical features and risk assessment of pre-diabetes in Spain: The prospective Mollerussa cohort study. BMJ Open 2017;7.*

(2) *Catalan M, Herreras Z, Pinyol M, Sala-Vila A, Amor AJ, Groot E de, Gilabert R, Ros E, Ortega E. Prevalence by sex of preclinical carotid atherosclerosis in newly diagnosed type 2 diabetes. Nutrition, Metabolism and Cardiovascular Diseases 2015;25:742–748.*

(3) *Carbonell M, Castelblanco E, Valldeperas X, Betriu À, Traveset A, Granado-Casas M, Hernández M, Vázquez F, Martín M, Rubinat E, Lecube A, Franch-Nadal J, Fernández E, Puig-Domingo M, Avogaro A, Alonso N, Mauricio D. Diabetic retinopathy is associated with the presence and burden of subclinical carotid atherosclerosis in type 1 diabetes. Cardiovasc Diabetol 2018;**17**:1–10.*

(4) *Alonso N, Traveset A, Rubinat E, Ortega E, Alcubierre N, Sanahuja J, Hernández M, Betriu A, Jurjo C, Fernández E, Mauricio D. Type 2 diabetes-associated carotid plaque burden is increased in patients with retinopathy compared to those without retinopathy. Cardiovasc Diabetol 2015;14:1–9.*

*Supplementary Table S1. Description of the analyses implemented.*

| Analysis | Subjects | Formula |
|---|---|---|
| SCA presence | T1D | $f_i \sim Sex + age + HT + DLP + BMI + waist + \boldsymbol{SCA\ presence} + HbA1c + glucose + sample\ origin + smoking + diabetes\ duration + Antiplatelet + ALT$ |
| SCA presence | T2D | $f_i \sim Sex + age + HT + DLP + BMI + waist + \boldsymbol{SCA\ presence} + HbA1c + glucose + sample\ origin + smoking + diabetes\ duration$ |
| SCA presence | CT | $f_i \sim Sex + age + HT + DLP + BMI + waist + \boldsymbol{SCA\ presence} + HbA1c + glucose + sample\ origin + smoking$ |
| SCA burden | T1D | $f_i \sim Sex + age + HT + DLP + BMI + waist + \boldsymbol{SCA\ burden} + HbA1c + glucose + sample\ origin + smoking + diabetes\ duration + Antiplatelet + ALT$ |
| SCA burden | T2D | $f_i \sim Sex + age + HT + DLP + BMI + waist + \boldsymbol{SCA\ burden} + HbA1c + glucose + sample\ origin + smoking + diabetes\ duration$ |
| SCA burden | CT | $f_i \sim Sex + age + HT + DLP + BMI + waist + \boldsymbol{SCA\ burden} + HbA1c + glucose + sample\ origin + smoking$ |
| SCA presence sub-group | T1D and T2D | $f_i \sim Sex + age + HT + DLP + BMI + waist + \boldsymbol{SCA\ presence} + HbA1c + glucose + sample\ origin + smoking + DM + diabetes\ duration + Antiplatelet + ALT + SCA\ presence{:}DM + \boldsymbol{SCA\ presence{:}subgroup}$ |
| SCA presence sub-group | CT | $f_i \sim Sex + age + HT + DLP + BMI + waist + \boldsymbol{SCA\ presence} + HbA1c + glucose + sample\ origin + smoking + \boldsymbol{SCA\ presence{:}subgroup}$ |
| SCA burden sub-group | T1D and T2D | $f_i \sim Sex + age + HT + DLP + BMI + waist + \boldsymbol{SCA\ burden} + HbA1c + glucose + sample\ origin + smoking + DM + diabetes\ duration + Antiplatelet + ALT + SCA\ burden{:}DM + \boldsymbol{SCA\ burden{:}subgroup}$ |
| SCA burden sub-group | CT | $f_i \sim Sex + age + HT + DLP + BMI + waist + \boldsymbol{SCA\ burden} + HbA1c + glucose + sample\ origin + smoking + \boldsymbol{SCA\ burden{:}subgroup}$ |

*HT, hypertension; DLP, dyslipidemia; BMI, body mass index; SCA, subclinical carotid atherosclerosis; HbA1c, glycated hemoglobin, DM, diabetes mellitus; ALT, Alanine aminotransferase levels. Subgroup represents each risk variable used in the contrast analysis, i.e., smoking habit, hypertension, dyslipidaemia, antiplatelet use and sex.*

**Contrast analysis:** For smoking-specific differences, multiple linear regression models were applied to each metabolite feature adding an interaction term between SCA presence or burden and smoking habit (No smoker = 0 and Former/current smoker = 1). Using this configuration, the p-value and regressor associated to presence/burden of SCA were assigned to non-smokers ($\beta_{\text{non-smoker}}$). The effect of SCA presence/burden in smokers ($\beta_{\text{smoker}}$) was computed by summing the regressor of the variable SCA presence/burden and the interaction between SCA presence/burden and smoking habit. Then, the t-value was computed dividing the effect ($\beta_{\text{smoker}}$) by its standard error and the p-value associated to SCA presence/burden in smokers was computed. Again, False Discovery Rate (FDR) was controlled using the R package qvalue. The same procedure was repeated to determine the rest of sub-group-specific differences associated to SCA presence and burden. In Supplementary Table S1, a detailed description of the analyses performed is provided.

# Supplemental Tables and supporting information - Lipidomic analysis reveals metabolism alteration associated with subclinical carotid atherosclerosis in type 2 diabetes

*Supplementary Table S2. Summary of lipid species identified using LipidSearch in positive acquisition mode. Values represent the mean (standard deviation) of their relative abundance per lipid class for all the population, subjects without diabetes, subjects with type 1 diabetes and type 2 diabetes.*

| Lipid class | Lipids | Total | Control | T1D | T2D |
|---|---|---|---|---|---|
| AcCa | 23 | 15.6504 (1.1630) | 15.6200 (1.1439) | 15.6368 (1.1788) | 15.7043 (1.1715) |
| Cer | 31 | 15.0622 (1.3304) | 15.0561 (1.3366) | 14.9234 (1.3758) | 15.2033 (1.2621) |
| CerG3GNAc 1 | 1 | 14.7634 (0.2902) | 14.7544 (0.2853) | 14.9134 (0.2467) | 14.6294 (0.2677) |
| ChE | 13 | 17.2875 (1.8992) | 17.2867 (1.9005) | 17.2865 (1.9389) | 17.2893 (1.8603) |
| Co | 1 | 15.0964 (0.3995) | 15.2322 (0.3665) | 15.0790 (0.3633) | 14.9170 (0.4081) |
| DG | 15 | 15.6459 (1.5055) | 15.6382 (1.4852) | 15.4054 (1.5153) | 15.8709 (1.4898) |
| GM3 | 4 | 14.7840 (0.5519) | 14.7739 (0.5308) | 14.7811 (0.6052) | 14.8003 (0.5248) |
| Hex1Cer | 8 | 14.9589 (1.0657) | 14.9742 (1.0615) | 15.0562 (1.0508) | 14.8430 (1.0759) |
| Hex2Cer | 3 | 16.0605 (0.5960) | 16.0809 (0.5942) | 16.1608 (0.5640) | 15.9355 (0.6081) |
| Hex3Cer | 1 | 15.1726 (0.3157) | 15.1409 (0.3023) | 15.3604 (0.2707) | 15.0319 (0.2861) |
| LPC | 55 | 16.4042 (2.0078) | 16.4230 (1.9994) | 16.5488 (2.0144) | 16.2382 (2.0006) |
| LPE | 8 | 14.6192 (1.0677) | 14.5823 (1.0726) | 14.6978 (1.0648) | 14.5920 (1.0608) |
| MePC | 43 | 17.1592 (2.3907) | 17.1958 (2.3694) | 17.1280 (2.3820) | 17.1404 (2.4271) |
| PC | 120 | 17.0603 (2.0103) | 17.0730 (1.9921) | 17.0643 (2.0207) | 17.0394 (2.0244) |
| PE | 28 | 16.1572 (1.3546) | 16.1098 (1.3531) | 16.2143 (1.3800) | 16.1646 (1.3296) |
| PI | 17 | 15.4816 (1.2519) | 15.5027 (1.2364) | 15.4195 (1.2697) | 15.5137 (1.2535) |
| SM | 70 | 17.1457 (2.0819) | 17.1662 (2.0742) | 17.1215 (2.0879) | 17.1416 (2.0861) |
| ST | 1 | 14.2100 (0.3108) | 14.1445 (0.2882) | 14.3595 (0.2769) | 14.1494 (0.3222) |
| SiE | 1 | 14.1977 (0.7080) | 14.1503 (0.5719) | 14.6668 (0.6394) | 13.8034 (0.6733) |
| TG | 66 | 17.0302 (2.0877) | 17.0272 (2.0905) | 16.9762 (2.0937) | 17.0865 (2.0768) |
| ZyE | 1 | 14.3213 (0.2939) | 14.3737 (0.2997) | 14.1423 (0.2126) | 14.4179 (0.2795) |

*Supplementary Table S3. Summary of lipid species identified using LipidSearch in negative acquisition mode. Values represent the mean (standard deviation) of their relative abundance per lipid class for all the population, subjects without diabetes, subjects with type 1 diabetes and type 2 diabetes.*

| Lipid class | Lipids | Total | Control | T1D | T2D |
|---|---|---|---|---|---|
| Cer | 14 | 14.9484 (1.1287) | 14.9742 (1.1271) | 14.8611 (1.1450) | 15.0034 (1.1088) |
| CerP | 11 | 14.4098 (1.1189) | 14.4142 (1.1084) | 14.4728 (1.1210) | 14.3354 (1.1279) |
| GM3 | 3 | 13.9847 (0.4036) | 13.9595 (0.3893) | 14.0683 (0.3819) | 13.9318 (0.4322) |
| Hex1Cer | 11 | 14.2450 (0.8336) | 14.2664 (0.8331) | 14.2788 (0.8640) | 14.1785 (0.7974) |
| Hex2Cer | 1 | 15.2328 (0.2901) | 15.2497 (0.2596) | 15.3612 (0.2477) | 15.0719 (0.2992) |
| Hex3Cer | 1 | 13.7767 (0.2342) | 13.7523 (0.2119) | 13.9042 (0.2000) | 13.6761 (0.2394) |
| LPA | 1 | 14.8373 (1.1603) | 15.0011 (0.9908) | 15.4027 (0.5886) | 13.9967 (1.3659) |
| LPC | 11 | 16.3196 (1.4546) | 16.3332 (1.4438) | 16.4163 (1.5133) | 16.1970 (1.3975) |
| LPE | 6 | 15.1843 (0.4471) | 15.1290 (0.4131) | 15.3292 (0.5052) | 15.1089 (0.3883) |
| LdMePE | 2 | 13.3037 (0.6468) | 13.3069 (0.6496) | 13.4972 (0.5201) | 13.0931 (0.6993) |
| PA | 1 | 13.7444 (0.1882) | 13.7382 (0.2003) | 13.7715 (0.1682) | 13.7245 (0.1886) |
| PC | 50 | 15.6312 (1.5933) | 15.6408 (1.5841) | 15.5972 (1.5899) | 15.6537 (1.6096) |
| PE | 26 | 15.2201 (0.9825) | 15.1634 (0.9784) | 15.2282 (0.9993) | 15.2924 (0.9652) |
| PI | 14 | 15.3496 (1.1831) | 15.3602 (1.1730) | 15.2985 (1.1911) | 15.3896 (1.1875) |
| SM | 1 | 14.2222 (0.3059) | 14.2496 (0.2791) | 14.1523 (0.3127) | 14.2573 (0.3244) |

*Supplementary Table S4. Number of significant LC-MS features in each analysis and ionization mode and for each group of subjects. Counts of significant features are shown in (positive acquisition mode, negative acquisition mode).*

| Risk-group | SCA presence | | | SCA burden | | |
|---|---|---|---|---|---|---|
| | T1D | T2D | Controls | T1D | T2D | Controls |
| **General** | (0, 0) | (26, 0) | (0, 0) | (33, 35) | (0, 0) | (2, 0) |
| **Non-smokers** | (0, 0) | (0, 0) | (0, 0) | (0, 1) | (0, 0) | (0, 0) |
| **Smokers** | (1, 0) | (66, 1) | (0, 0) | (6, 14) | (40, 109) | (3, 0) |
| **HT No** | (0, 0) | (0, 0) | (0, 0) | (2, 6) | (0, 0) | (0, 0) |
| **HT Yes** | (0, 0) | (0, 0) | (0, 0) | (0, 1) | (0, 0) | (1, 3) |
| **DLP No** | (0, 0) | (4, 0) | (0, 0) | (1, 15) | (53, 4) | (0, 0) |
| **DLP Yes** | (0, 0) | (0, 0) | (0, 0) | (0, 1) | (0, 0) | (0, 0) |
| **Antiplatelet No** | (0, 0) | (0, 0) | - | (5, 28) | (0, 0) | - |
| **Antiplatelet Yes** | (0, 0) | (0, 0) | - | (0, 1) | (0, 0) | - |
| **Men** | (0, 0) | (6, 0) | (0, 0) | (0, 1) | (0, 0) | (0, 0) |
| **Women** | (0, 0) | (0, 0) | (0, 0) | (9, 25) | (0, 4) | (1, 0) |
